# Complete Genome Sequence of *Klebsiella pneumoniae* Carbapenemase-Producing *K. pneumoniae* Siphophage Sushi

**DOI:** 10.1128/genomeA.00994-15

**Published:** 2015-09-03

**Authors:** Dat T. Nguyen, Lauren E. Lessor, Jesse L. Cahill, Eric S. Rasche, Gabriel F. Kuty Everett

**Affiliations:** Center for Phage Technology, Texas A&M University, College Station, Texas, USA

## Abstract

*Klebsiella pneumoniae* is a Gram-negative bacterium in the family *Enterobacteriaceae*. It is associated with numerous nosocomial infections, including respiratory and urinary tract infections in humans. The following reports the complete genome sequence of *K. pneumoniae* carbapenemase-producing *K. pneumoniae* T1-like siphophage Sushi and describes its major features.

## GENOME ANNOUNCEMENT

*Klebsiella pneumoniae* is an opportunistic pathogen that is commonly found in the environment (1). Due to the emergence of multidrug-resistant strains, including *K. pneumoniae* carbapenemase (KPC)-producing strains, nosocomial infections caused by *Klebsiella* species are a leading cause of morbidity and mortality (2). With limited clinical treatment options for *K. pneumoniae* KPC strains, there is an urgent need for alternative treatments. The use of bacteriophages may be an option for the biocontrol and treatment of *K. pneumoniae* infections. Here, we describe the complete genome of Sushi, a T1-like siphophage active against KPC-producing *K. pneumoniae* strain A1.

Bacteriophage Sushi was isolated from a sewage sample collected in College Station, Texas, USA. Phage DNA was sequenced in an Illumina MiSeq 250-bp paired-end run with a 550-bp insert library at the Genomic Sequencing and Analysis Facility at the University of Texas (Austin, TX, USA). Quality-controlled, trimmed reads were assembled to a single contig of circular assembly at 30.9-fold coverage using SPAdes version 3.5.0. The contig was confirmed to be complete by PCR using primers that face the upstream and downstream ends of the contig. Products from the PCR amplification of the junctions of concatemeric molecules were sequenced by Sanger sequencing (Eton Bioscience, San Diego, CA, USA). Genes were predicted using GeneMarkS (3) and corrected using software tools available on the Center for Phage Technology (CPT) Galaxy instance (https://cpt.tamu.edu/galaxy-pub). The morphology of phage Sushi was determined using transmission electron microscopy performed at the Texas A&M University Microscopy and Imaging Center.

Sushi has a 48,754-bp genome with a G+C content of 50.8%. The G+C content for Sushi is slightly lower than that of its host at 57.5% (4). The Sushi genome has a coding density of 92.4% and 76 coding sequences, of which 28 have an annotated gene function predicted by BLASTp and InterPro Scan analysis (5, 6). Sushi has shared nucleotide sequence identity with *Klebsiella* phage KP36 (GenBank accession no. JF501022, 48.7% identity); *Enterobacteria* phage T1 (NCBI reference sequence NC_005833, 47.3% identity); and *Escherichia* phage TLS (NCBI reference sequence NC_009540, 46.0%) as determined by Emboss Stretcher (7). It is a member of the Lytic1 cluster of phages described by Grose and Casjens (8). The TerL protein of Sushi shows homology to the TerL of phages that use a pac-headful DNA packaging mechanism. For annotation purposes, Sushi has been opened to the *terS* gene (9).

Sushi contains T1-like core genes encoding proteins involved in DNA replication, DNA packaging, morphogenesis, recombination, and lysis. Sushi encodes a DNA *N*-6-adenine methyltransferase to protect its DNA from host restriction enzymes (10). In addition, Sushi encodes a TLS-like C-5 cytosine-specific DNA methylase. Like T1, the lysis cassette of Sushi consists of a pinholin (class II, two transmembrane domains in an N-in C-in topology), a SAR endolysin, and a unimolecular spanin (11–13).

### Nucleotide sequence accession number. 

The genome sequence of phage Sushi was contributed to GenBank under the accession number KT001920.
